# Adrenal function in patients receiving rifampin-based anti-tuberculosis regimens: A cross-sectional study in Iran

**DOI:** 10.22088/cjim.11.4.458

**Published:** 2020

**Authors:** Sirous Jafari, Javad Behjati, Kaveh Shafaei, Seyed Ali Dehghan Manshadi, Arash Seifi

**Affiliations:** 1 Department of Infectious Diseases, Faculty of Medicine, Tehran University of Medical Sciences, Tehran, Iran; 2 Department of Endocrinology, Faculty of Medicine, Tehran University of Medical Sciences, Tehran, Iran

**Keywords:** Adrenal Gland Diseases, Tuberculosis, Adrenocorticotropic Hormone

**Dear Sir,**

Tuberculosis (TB) is still a major cause of morbidity and mortality in many parts of the world including Iran. Acid-fast bacillus *Mycobacterium tuberculosis* is the causative agent and often involves the respiratory system; however, in one-third of the cases, other organs are infected. In spite of availability of anti-TB drugs, death and disability caused by the disease are still high (1).

Adrenal insufficiency is a potentially life-threatening consequence of TB which is commonly underdiagnosed; specifically in the developing countries where infections are the major cause of the condition. TB adrenalitis is a rare cause of adrenal insufficiency more reported in developing countries. On the other side, rifampin as part of the standard anti-TB treatment is potentially an inducer of liver enzymes and may facilitate cortisol catabolism and hence, cortisol deficiency (2). Based on these considerations, a cross-sectional (descriptive-analytic) study was conducted. All patients with the definitive diagnosis of TB recruited in a central referral hospital from 2014 to 2015 were included. They received rifampin-based regimens. Adrenal function tests (serum cortisol and ACTH) were evaluated at baseline and one week after starting treatment. The results were compared using Student’s t-test. Thirty patients participated, from whom 7 were not considered in final analysis due either to early discharge or death.

In total, 23 patients were included in the study; 19 (82.6%) men and 4 (17.4%) women. The mean age of the participants was 41.83 years (minimum: 22; maximum: 84; SD=14.81). Most participants were Iranian (78.3). The mean weight of all patients was 59.39 (SD=8.44); and the mean height was 172.65 (SD=6.11). The mean body mass index (BMI) of all patients was 19.52. Most common form of the disease was pulmonary tuberculosis (43.5%).

The normal range of serum cortisol level was considered to be from 5 to 25 μg /dL. The mean serum cortisol level was 26.13 (SD=14.05) μg/dl at baseline, while 27.04 (SD=17.20) μg/dL on day 7. There was no significant difference in the mean cortisol level at baseline and on day 7 based on the paired t-test (P>0.05). Normal ACTH range was defined from 7 to 63 pg/ml. The mean ACTH level was 13.75 (SD=7.21) pg/ml for the patients before initiation of treatment; on day 7 the mean was reported to be 16.92 (SD=7.86) pg/ml. A significant difference was observed in the mean serum ACTH level at baseline and on the seventh day of treatment; with higher ACTH levels on day 7 (p<0.05).

Independent t-test showed that there was a correlation between serum cortisol levels after treatment and the patients' final status (alive vs. dead) (P = 0.03). Although, no notable correlation was found for ACTH level, the mean serum ACTH level in patients who died was significantly higher at baseline [21.53 (SD=12.08) at baseline and 15.98 (SD=7.75) after treatment, respectively (P = 0.002)].

Uedney et al. showed that serum cortisol levels had no significant difference in patients and controls, but the 30 minute cortisol level to the ACTH stimulation test showed a significant reduction in patients with tuberculosis compared to controls, indicating subclinical adrenocortical failure in 23% of patients (3). We also assume that stimulation tests in our patient population would probably show this reduced response. In a study by Krishnaet al. adrenocorticular storage in patients with active TB was significantly less than that of the control group (4). The increased levels of ACTH and cortisol during treatment can be related to the extent of the involvement. The possibility that increased cortisol metabolism has led to more ACTH secretion to compensate cortisol should also be taken into account. A limitation of the study could be the short follow-up of the patients. Designing long-term cohorts may help with better understanding the effects of TB on hypothalamo-pituitary-adrenal (HPA) axis both at baseline and with anti-TB agents such as rifampin.

We suggest that the insignificant cortisol increments may potentially lead to adrenal insufficiency in long-term; hence, monitoring of adrenal function in long-term treatment of TB seems essential.

## Conflict of Interest:

None of the authors have any conflicts of interest to report.
